# Role of the lncRNA ABHD11-AS_1_ in the tumorigenesis and progression of epithelial ovarian cancer through targeted regulation of RhoC

**DOI:** 10.1186/s12943-017-0709-5

**Published:** 2017-08-17

**Authors:** Dan-Dan Wu, Xi Chen, Kai-Xuan Sun, Li-Li Wang, Shuo Chen, Yang Zhao

**Affiliations:** Department of Gynecology, the First Affiliated Hospital of China Medical University, No.155 Nanjing North Street, Heping Area, Liaoning Shenyang, 110001 People’s Republic of China

**Keywords:** lncRNA ABHD11-AS_1_, Tumor, Epithelial ovarian cancer, RhoC, Tumorigenesis and progression

## Abstract

**Background:**

There is increasing evidence in support of the role of lncRNAs in tumor cell proliferation, differentiation and apoptosis.

**Methods:**

We examined the expression of the lncRNA ABHD11-AS_1_ in epithelial ovarian cancer (EOC) tissues and normal ovarian tissues by real-time quantitative PCR (qRT-PCR). After inducing ABHD11-AS_1_ downregulation by small interfering RNA (siRNA) or ABHD11-AS_1_ overexpression by plasmid transfection, we examined the EOC cell phenotypes and expression of related molecules.

**Results:**

Expression of the lncRNA ABHD11-AS_1_ in EOC tissues was higher than that in normal ovarian tissue. It was positively associated with the tumor stage (stage I/II vs. stage III/IV), and it was lower in the well-differentiated group than in the poorly/moderately differentiated group. Overexpression of ABHD11-AS_1_ in the ovarian cancer cell lines A2780 and OVCAR3 promoted ovarian cancer cell proliferation, invasion and migration, and inhibited apoptosis. Silencing of ABHD11-AS_1_ had the opposite effect. Subcutaneous injection of tumor cells in nude mice showed that ABHD11-AS_1_ could significantly promote tumor growth. In addition, intraperitoneal injection of tumor cells in the nude mice resulted in an increase in the metastatic ability of the tumor. Further, overexpression of ABHD11-AS_1_ upregulated the expression of RhoC and its downstream molecules P70s6k, MMP2 and BCL-xL. Silencing of ABHD11-AS_1_ had the opposite effect. The RNA pull-down assay showed that ABHD11-AS_1_ can combine directly with RhoC. Silencing of RhoC was found to inhibit the cancer-promoting effects of lncRNA ABHD11-AS_1_. Thus, it seems that RhoC is a major target of the lncRNA ABHD11-AS_1_.

**Conclusions:**

This is the first study to demonstrate the role of RhoC in the tumor-promoting effects of the lncRNA ABHD11-AS_1_. The present findings shed light on new therapeutic targets for ovarian cancer treatment.

**Electronic supplementary material:**

The online version of this article (doi:10.1186/s12943-017-0709-5) contains supplementary material, which is available to authorized users.

## Background

Ovarian cancer is a malignant tumor of the female reproductive organs that accounts for a third of all gynecological malignant tumors. Because of its hidden onset, poor prognosis and high fatality rate, the overall survival rate of this cancer is not satisfactory [1]. Therefore, there is need to identify effective biomarkers by studying the mechanism of tumorigenesis of ovarian cancer and its progression, as such biomarkers may improve the clinical diagnosis and treatment of ovarian cancer.

In recent years, long noncoding RNAs (lncRNAs) have been found to play a role in the occurrence, invasion, metastasis and drug resistance of tumors [2–6]. LncRNAs are non-encoding RNA molecules that are made up of more than 200 nucleotides [7]; they regulate gene expression and participate in several biological functions through a variety of pathways and molecular mechanisms. Anomalies in the expression and regulation of lncRNA are closely related to the development of many malignant tumors; therefore, lncRNAs are believed to play an important role in cell proliferation, differentiation and apoptosis. However, more evidence is needed to confirm these findings. In particular, there is very little research on the role of the lncRNA ABHD11-AS_1_ (ABHD11 antisense RNA1), which is found in human chromosome 7 q11. 23. Lin [8] showed that ABHD11-AS_1_ expression was significantly higher in gastric cancer tissues than in normal gastric tissues, which implied that this lncRNA might function as a cancer biomarker. Yang [9] also showed that ABHD11-AS_1_ expression was significantly higher in the gastric juice of patients with gastric cancer than in normal patients, but the mechanism of action of this cancer-related lncRNA is unclear. Moreover, there are no reports about the role or mechanism of the lncRNA ABHD11-AS_1_ in ovarian cancer.

Ras homolog gene family member C (RhoC) is a vital RhoGTPase involved in cytoskeleton reorganization and cell adhesion and migration, especially in the regulation of ovarian cancer tumorigenesis and progression [10, 11]. Our previous studies found that RhoC expression was positively associated with many oncogenes in ovarian cancer, such as MMP2, BCL-xL and P70s6k [12]. RhoC can stimulate the expression of MMP2, which promotes cancer cell invasion and metastasis through degradation of the extracellular matrix [13]. BCL-xL, a member of the BCL-2 family, exhibits anti-apoptotic effects and has also been found to be upregulated in bladder cancer [14, 15]. P70s6k (P70 ribosomal S6 kinase), a downstream effector of PI3K/AKT signaling pathway, has been reported to participate in protein synthesis and promote aggressiveness and metastasis in ovarian cancer [16, 17]. Based on all these findings, it would be interesting to investigate whether RhoC plays a role in the development and progression of ovarian cancer.

The present study is the first one to investigate the role and mechanism of the lncRNA ABHD11-AS_1_ in ovarian cancer occurrence and development. We also explored whether the effects of ABHD11-AS_1_ were brought about by its interaction with RhoC, as well as its downstream molecules.

## Methods

### Ovarian cancer specimens

Fifty-one epithelial ovarian cancer tissue and 13 normal ovarian tissue specimens were collected from patients who had undergone surgical resection at the Department of Gynecology of the First Affiliated Hospital of China Medical University (Shenyang, China). Two pathologists confirmed the tumor specimens independently. Samples were frozen immediately in liquid nitrogen and stored at −80 °C until use. None of the patients had undergone preoperative chemotherapy or radiotherapy. Informed consent was obtained from all the subjects. The China Medical University Ethics Committee approved of the study, and all the specimens were handled and anonymized according to ethical and legal standards (No: 2014–27).

### Cell culture and transfection

The human ovarian carcinoma cell lines A2780 and OVCAR3 were purchased from Jennio Biotech Co. Ltd. (GuangZhou, China). The human cell lines A2780 cells were cultured in Dulbecco’s modified Eagle’s medium (DMEM; HyClone, Logan, UT, USA) and the OVCAR3 cells were cultured in RPMI 1640 (HyClone) supplemented with penicillin/streptomycin (100 U/mL) in 10% fetal bovine serum (FBS). The cells were cultured in an incubator at 37 °C in a 5% CO_2_ atmosphere. All transfection experiments were carried out using Lipofectamine 2000 according to the manufacturer’s instructions (Invitrogen, Carlsbad, USA). The ABHD11-AS_1_ siRNA sequences were as follows: 5′-GCUACGAGAUCAUGAGCCAdTdT-3′ (sense) and 5′-UGGCUCAUGAUCUCGUAGCdTdT-3′ (anti-sense). The target sequences of RhoC siRNA were 5′-GUGCCUUUGGCUACCUUGAdTdT-3′ (sense) and 5′-UCAAGGUAGCCAAAGGCACdTdT-3′ (anti-sense). The ABHD11-AS_1_ sequences are shown in Additional file 1: Table S3.

### Tetrazolium assay

Cells were seeded in 96-well plates at a density of 3000 cells per well. At 0 h, 24 h, 48 h, and 72 h after seeding, the cells were incubated with 20 μL of 5 mg/mL tetrazolium (MTT) solution (Solarbio, Beijing, China) at 37 °C for 4 h. Then, the medium was removed, and the precipitated formazan was dissolved in 150 μL dimethyl sulfoxide (DMSO). After shaking for 10 min, the absorbance at 490 nm was determined using a microplate spectrophotometer (BioTek Instruments, Winooski, VT, USA), and was used as an indicator of cell proliferation.

### Apoptosis assay

Cells were collected 48 h after transfection and washed twice with cold phosphate-buffered saline (PBS). For the ABHD11-AS_1_ plasmid transfection, cell apoptosis was quantified using 7AAD and PE-labeled Annexin V (BD Biosciences) with flow cytometry, according to the manufacturer’s protocol. For siRNA transfection, cell apoptosis was quantified using annexin V–FITC and PI (BD Biosciences) with flow cytometry. The apoptosis rate was determined by flow cytometry within 1 h.

### Wound healing assay

Cells were cultured to 80% confluence in 6-well culture plates. Then, the monolayers were scratched with a 200-μL pipette tip. The cells were washed with PBS and cultured in FBS-free medium with mitomycin C (20 μg/ml). Wounds were observed under a light microscope and photographed at 0 h, 24 h, and 48 h. The wound areas were measured using the ImageJ software (National Institutes of Health, Bethesda, MD, USA). The wound healing rate was determined as follows: (area of original wound − area of wound at different times)/area of original wound × 100%. The wound healing rate was considered as an indicator of the migration ability of the cells.

### Invasion assay

We used Matrigel-coated Transwell cell culture chambers (BD Bioscience, San Jose, CA, USA) for the invasion assay. Filters were coated with 30 μL of basement membrane Matrigel (1:10). Cells (5 × 10^4^/L) resuspended in 200 μL serum-free medium were layered in the top compartment of the Transwell inserts. The bottom chambers contained 600 μL of complete medium that served as the chemoattractant. After 48 h of incubation at 37 °C, cells on the upper surface of the filter were removed using a cotton swab. The cells that had invaded the bottom of the top chamber were fixed with formaldehyde, stained with crystal violet, and counted under an Olympus fluorescence microscope (Tokyo, Japan).

### qRT-PCR

Total RNA was isolated from ovarian carcinoma cell lines and tissues with TRIzol reagent (Takara, Shiga, Japan), and OD260/280 was measured with a spectrophotometer (Unico, Shanghai, China). An OD260/280 value of 1.8–2.0 indicated that the RNA quality was good. Then, 2 μg of RNA was reverse-transcribed to complementary DNA (cDNA) using the avian myeloblastosis virus transcriptase and random primers (Takara) according to the manufacturer’s protocol. Then, the cDNA was amplified by real-time quantitative PCR with the SYBR Premix Ex Taq™ II kit (Takara, Shiga, Japan). The relative expression of the target genes was determined by comparing the threshold cycle (Ct) of the target genes to that of 18S rRNA (18 s) using the 2^-ΔΔCt^ method (GenePharma).

### Western blotting

The complete endometrial carcinoma proteome was extracted in radio-immunoprecipitation assay buffer, which prevented protease-mediated sample degradation. The protein concentration was determined for each sample. Then, 40 μg of the denatured proteome was resolved by 10 or 12% sodium dodecyl sulfate (SDS)-polyacrylamide gel electrophoresis and then electrotransferred to Hybond membranes (Amersham, Munich, Germany). After the membranes were blocked with 5% fat-free milk at room temperature for 2 h, they were incubated with primary antibodies against RhoC (1:500; Santa Cruz Biotechnology, Santa Cruz, CA, USA), P70S6K, MMP2, and Bcl-xL (1:1000; Proteintech, Proteintech Group, USA) at 4 °C overnight. The membranes were washed three times with Tris-buffered saline containing Tween-20 (TBST), and then anti-rabbit or anti-goat secondary antibodies (1:5000) were added. After 2 h of incubation at room temperature, the protein bands were visualized by enhanced chemiluminescence according to the manufacturer’s instructions (Santa Cruz Biotechnology, Santa Cruz, CA, USA). β-actin (1:3000; Proteintech, Proteintech Group, USA) served as the loading control.

### RNA pull-down assay

OVCAR3 normal and ABHD11-AS_1_-overexpressing cells were collected and lysed using a protein lysis buffer. Streptavidin magnetic beads were used to capture the biotin-labeled ABHD11-AS_1_ probe (Beijing Dingguo Changsheng biotech CO. China) according to the manufacturer’s protocol. Then, the biotinylated nucleic acid compounds were incubated with the protein extract of cells in a 42 °C water bath for 40 min. After elution of the magnetic beads, the protein samples were detected by western blotting analysis, with the extracted protein as the positive control and the antisense RNA as the negative control.

### In vivo tumorigenesis model

An in vivo model of ovarian cancer was established by subcutaneously and intraperitoneally injecting 5-week-old female BALB/c nude mice with 1 × 10^7^ A2780 cells transfected with lncRNA ABHD11-AS_1_ (or mock transfected) suspended in PBS (8 mice per group). The tumor volumes of the mice were measured every 3 days. Tumor volume was assessed by measuring the length (L) and width (W) of the tumor with calipers (tumor volume [mm^3^] = 0.5 × L × W^2^). After 4 weeks, the mice were euthanized, and the subcutaneous implanted tumors as well as the intraperitoneal metastatic lesions were excised, measured and photographed. All mice were obtained from Vital River Laboratories (Beijing, China) and housed in a specific pathogen-free environment. This experiment was conducted in accordance with the National Institutes of Health Guide for the Care and Use of Laboratory Animals and approved by the China Medical University Animal Care and Use Committee.

### Immunohistochemistry

Consecutive tissue sections were deparaffinized with xylene, rehydrated with alcohol, and subjected to antigen retrieval by heating in a target retrieval solution (Dako) for 15 min in a microwave oven (Oriental Rotor). The sections were quenched with 3% hydrogen peroxide for 20 min to block endogenous peroxidase activity. Nonspecific binding was prevented by adding 5% bovine serum albumin for 5 min. The sections were incubated for 15 min with the RhoC antibody and then incubated with HRP-conjugated anti-rabbit antibodies (Dako) for 15 min in a microwave oven. After each treatment, the slides were washed three times with TBST for 5 min, and the binding sites were visualized with 3, 3′-diaminobenzidine. After the sections were counterstained with Mayer’s hematoxylin, they were dehydrated, cleared, and mounted. Negative controls were prepared by omitting the primary antibody.

### Statistical analysis

Data are presented as the mean ± SD values, and were analyzed using the SPSS 18.0 software (SPSS Inc., Chicago, IL, USA). The unpaired two-tailed Student’s *t*-test, Mann–Whitney U-test, and Spearman’s correlation test were used to compare the two groups. All *p*-values are two-sided; *p* < 0.05 is indicative of statistical significance.

## Results

### Correlation of ABHD11-AS_1_ expression with the tumorigenesis and progression of epithelial ovarian carcinoma

**Fig. 1:**
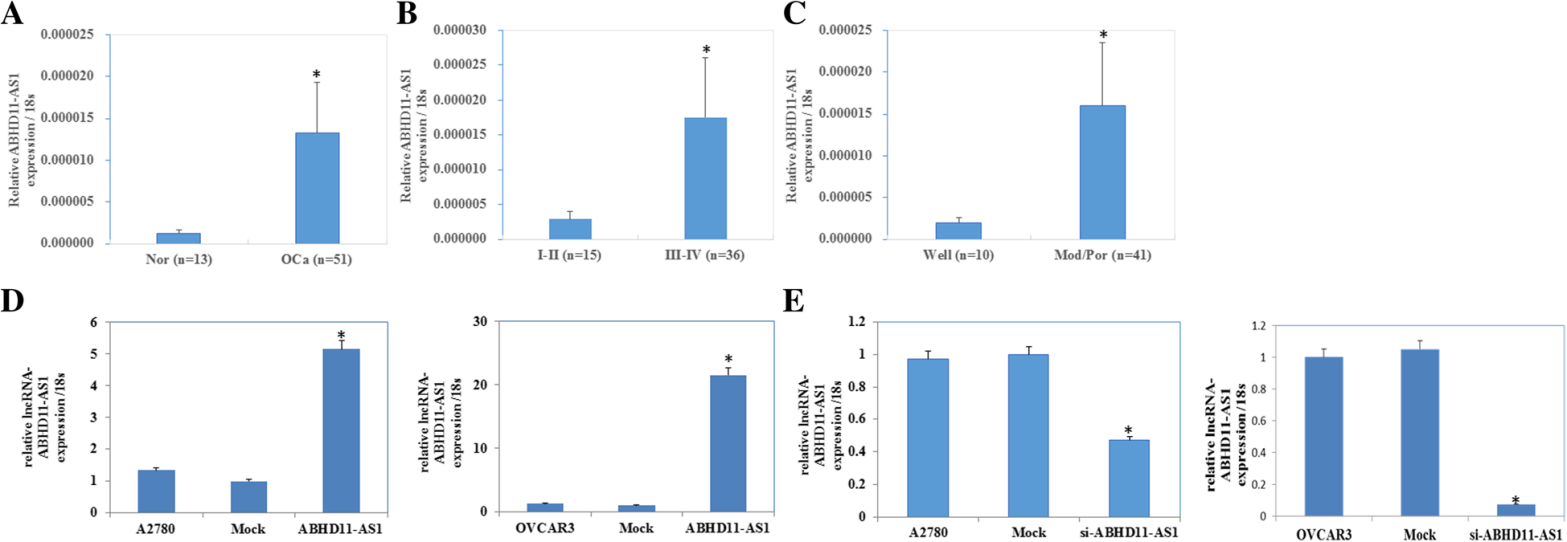
Correlation of the lncRNA ABHD11-AS_1_ expression with the pathogenesis and aggressiveness of ovarian carcinoma and efficiency of lncRNA ABHD11-AS_1_ transfection in ovarian carcinoma cell lines. lncRNA ABHD11-AS_**1**_ expression was significantly higher in epithelial ovarian carcinoma than in normal ovarian tissues (**a**). lncRNA ABHD11-AS_1_ expression was positively associated with the tumor stage according to the International Federation of Gynecology and Obstetrics (FIGO) staging system (stage I/II vs. stage III/IV) (**b**), and it was lower in the well-differentiated group than in the poorly/moderately differentiated group (well vs. poor /moderate) (**c**). *P < 0.05. After lncRNA ABHD11-AS_1_ transfection, the ovarian carcinoma cell lines exhibited significantly higher ABHD11-AS_1_ expression (**d**). Silencing of ABHD11-AS1 resulted in lower lncRNA ABHD11 expression (**e**)

ABHD11-AS_1_ expression was quantified in ovarian cancer tissues and normal ovarian tissue**s** using quantitative reverse transcription PCR (qRT-PCR). ABHD11-AS_1_ expression was significantly higher in ovarian cancer tissues than in normal ovarian tissue**s** (Fig. 1a, *p* < 0.05). Further, in the ovarian cancer tissues**,** it was positively associated with the tumor stage according to the International Federation of Gynecology and Obstetrics (FIGO) staging system (stage I/II vs. stage III/IV, Fig. 1b, *p* < 0.05), and it was lower in the well-differentiated group than in the poorly/moderately differentiated group (well vs. poor/moderate, Fig. 1c, *p* < 0.05; details provided in Additional file 1: Table S1 and S2).

### Effect of lncRNA ABHD11-AS1 transfection on in vitro ovarian cancer cell proliferation**,** apoptosis and aggressiveness

**Fig. 2:**
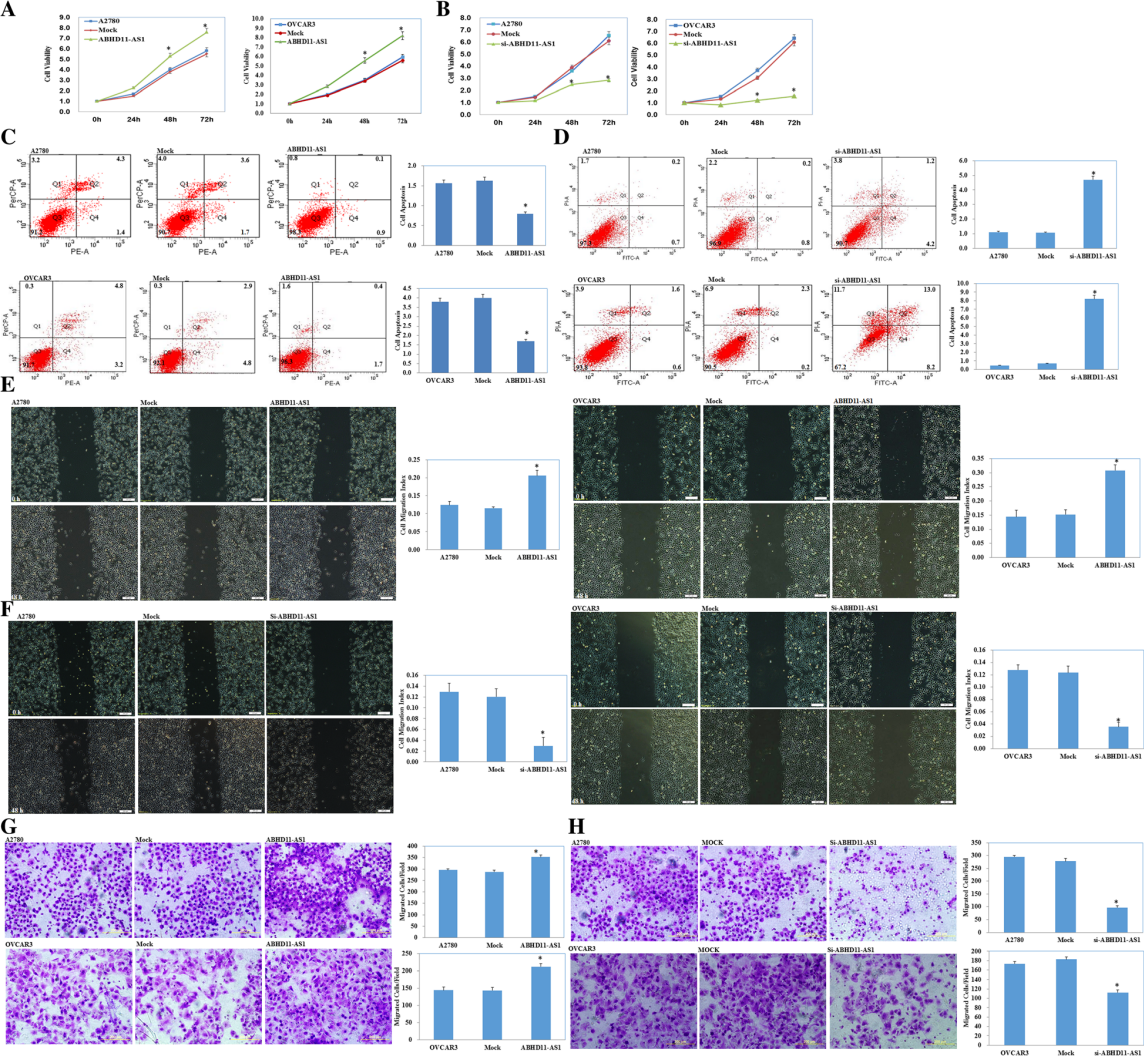
Effect of lncRNA ABHD11-AS_1_ transfection on the in vitro proliferation, apoptosis, and metastatic and invasive ability of ovarian carcinoma cell lines. After lncRNA ABHD11-AS_1_ transfection, the ovarian carcinoma cell lines exhibited significantly faster growth, decrease in apoptosis, and enhanced migration and invasion ability, compared with the control and mock cells (**a**, **c**, **e** and **g**). Silencing of the lncRNA ABHD11-AS1 resulted in the opposite effect (**b**, **d**, **f** and **h**). The results are representative of three separate experiments; data are expressed as the mean ± standard deviation values. * *P* < 0.05

The lncRNA ABHD11-AS_1_ was highly expressed in the A2780 and OVCAR3 cell lines after lncRNA ABHD11-AS_1_ transfection (Fig. 1d, *p* < 0.05). A2780 and OVCAR3 cells transfected with lncRNA ABHD11-AS_1_ exhibited significant rapid growth (Fig. 2a, *p* < 0.05), a decrease in cell apoptosis (Fig. 2c, *p* < 0.05), and an increase in the migration (Fig. 2e, *p* < 0.05) and invasion rate (Fig. 2g, *p* < 0.05), compared to the control and mock-transfected cells. Silencing of ABHD11-AS_1_ yielded opposite results (Figs. 1e, and, 2b–h, *p* < 0.05).

### Co-immunoprecipitation of ABHD11-AS_1_ with RhoC

**Fig. 3:**
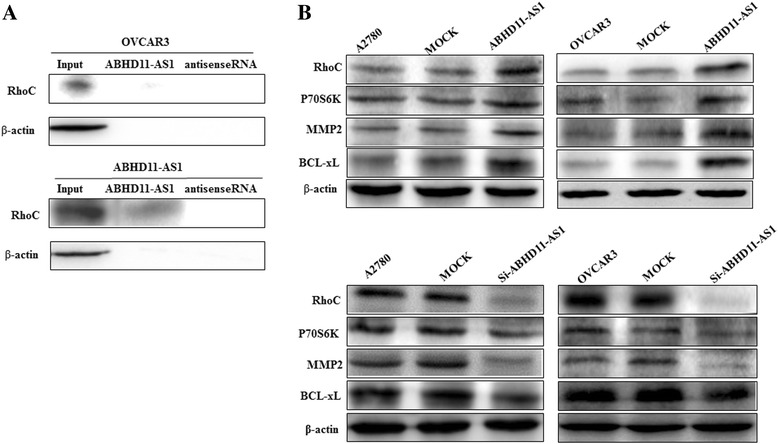
Effect of lncRNA ABHD11 transfection on ovarian carcinoma cell genotype in vitro. The results of the RNA pull-down assay demonstrated that expression of the RhoC protein pulled down by the ABHD11-AS1 probe was higher in the ABHD11-AS_1_-overexpressing cells than in the normal cells (**a**). lncRNA ABHD11-AS_1_ overexpression resulted in an increase in RhoC, p70s6k, MMP2, and BCL-xL expression compared with the mock and control groups (**b**). After silencing of the lncRNA ABHD11-AS_1_, RhoC, p70s6k, MMP2, and BCL-xL expression was also found to be decreased. *P < 0.05

RNA pull-down assay was used to detect the expression of RhoC, which was pulled down by biotin-labeled ABHD11-AS_1_. The results showed that the expression level of the RhoC protein was higher in the ABHD11-AS_1_-overexpression group than in the normal cell group. It was further proved that the lncRNA ABHD11-AS_1_ could directly combine with RhoC (Fig. 3a).

### Effect of lncRNA ABHD11-AS_1_ transfection on the in vitro ovarian carcinoma cell genotype

Western blot analysis demonstrated that lncRNA ABHD11-AS_1_ overexpression upregulated the expression of the RhoC protein and its downstream molecules P70s6k, MMP2 and BCL-xL (Fig. 3b). Silencing of lncRNA ABHD11-AS_1_ yielded opposite results (Fig. 3c).

### Effect of the lncRNA ABHD11-AS_1_ on in vivo tumor growth and metastasis

**Fig. 4:**
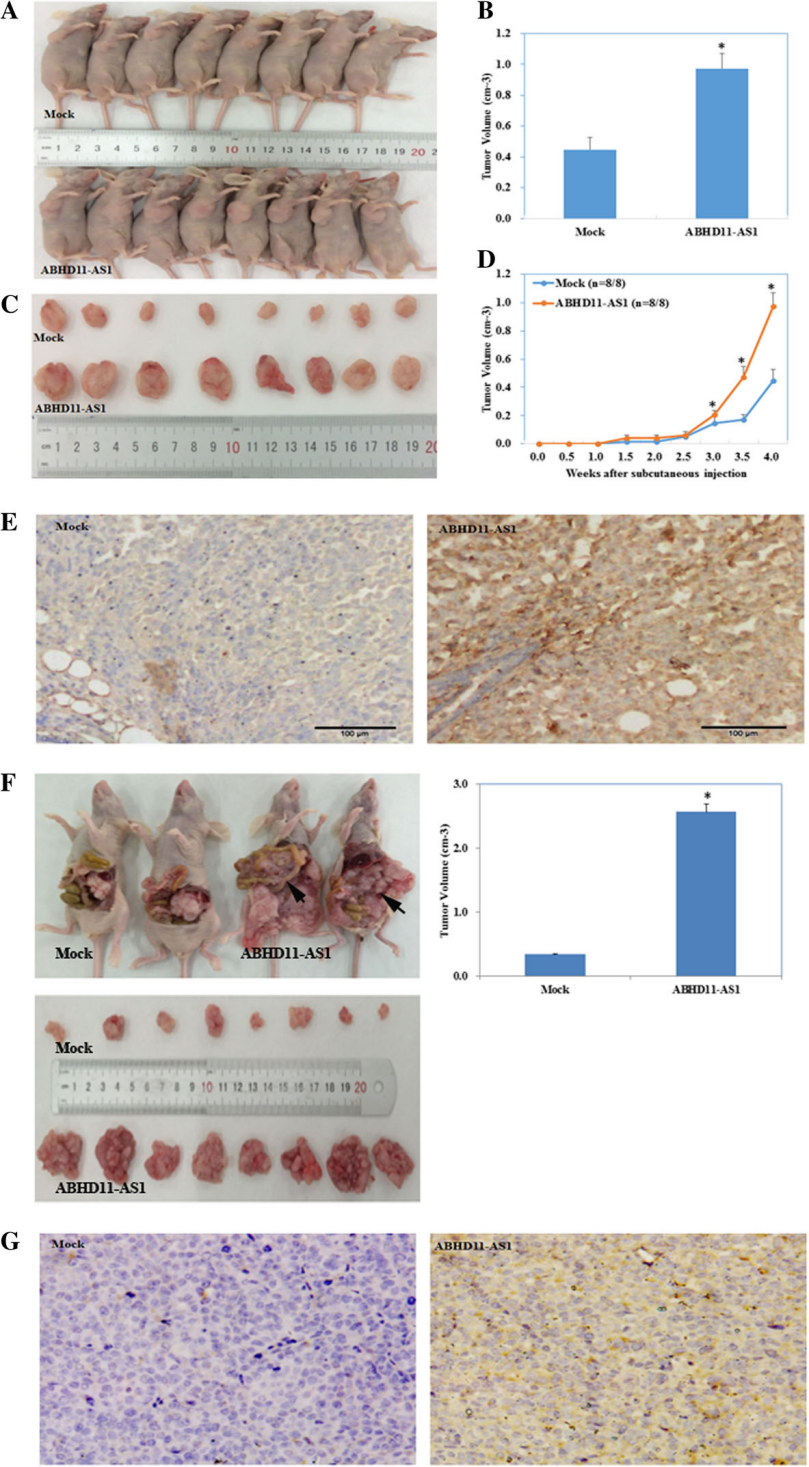
Effect of the lncRNA ABHD11 on in vivo tumor growth and metastasis ability as well as RhoC expression. The tumor xenograft volume in nude mice transfected with the lncRNA ABHD11-AS_1_ was greater than that in the mock nude mice (**a**, **b** and **c**). Tumor xenograft growth in the ABHD11-AS_1_-treated nude mice was faster than that in the control group (**d**). *P < 0.05. Macroscopic observation showed that the size and range of metastatic lesions were greater in the lncRNA ABHD11-AS_1_-transfected nude mice (**f**). Immunohistochemical analysis demonstrated a significant increase in RhoC expression in the ABHD11-AS_1_-transfected tumors compared with the control tumors both in the subcutaneously (**e**) and intraperitoneally injected nude mice (**g**). *P < 0.05

The tumor xenograft volume in nude mice transfected with the lncRNA ABHD11-AS_1_ was significantly greater than that in the mock nude mice (Fig. 4a–c *p* < 0.05), and the tumor growth rate was also significantly higher in the ABHD11-AS_1_-transfected mice (Fig. 4d, *p* < 0.05). Macroscopic observation of the intraperitoneally injected nude mice revealed that the size and total number of tumor nodes were higher in the ABHD11-AS_1_-transfected mice than in the mock nude mice. The metastatic lesions were significantly widespread among mesentery in the ABHD11-AS_1_-transfected group (Fig. 4f).

### Effect of the lncRNA ABHD11-AS_1_ on tumor progression via regulation of RhoC expression

**Fig. 5:**
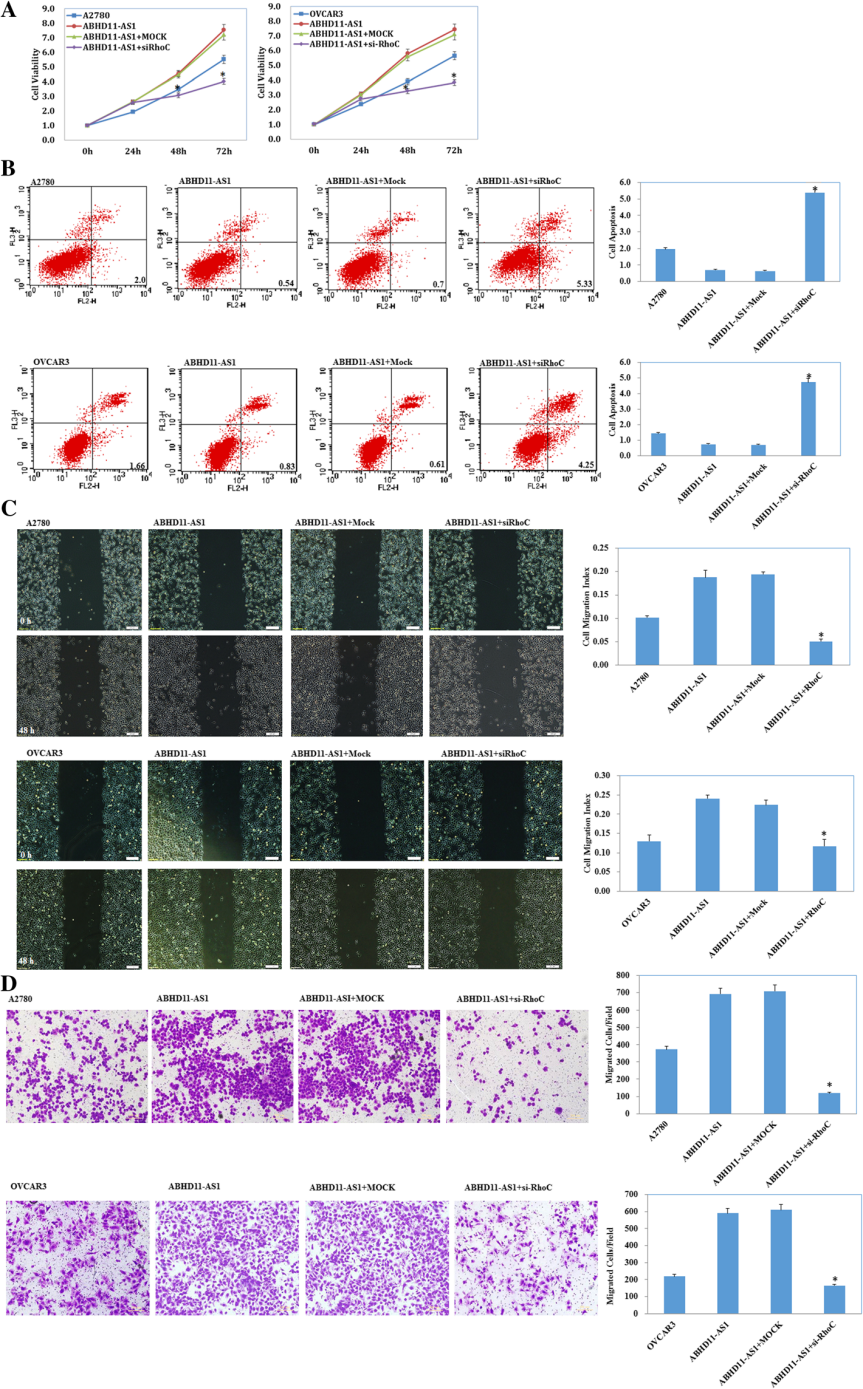
Effects of RhoC silencing on ovarian carcinoma cells transfected with the lncRNA ABHD11-AS_1_. After RhoC silencing, the ABHD11-AS_1_-overexpressing ovarian carcinoma cell lines exhibited significantly slower growth (**a**), migration (**c**), and invasion (**d**) and increased apoptosis (**b**) compared with the control and mock cells. The results are representative of three separate experiments; data are expressed as the mean ± standard deviation values. * *P* < 0.05

**Fig. 6:**
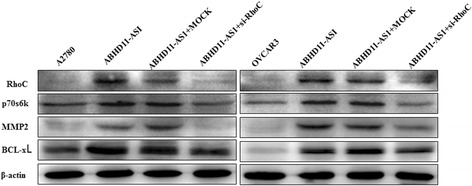
Effects of RhoC silencing on ABHD11-AS_1_-overexpressing ovarian carcinoma cells in vitro. RhoC silencing in ABHD11-AS_1_-overexpressing ovarian carcinoma cells resulted in a decrease in RhoC, p70s6k, MMP2, and BCL-xL expression

Immunohistochemical analysis demonstrated that the expression of RhoC in the subcutaneous xenograft of nude mice in the lncRNA ABHD11-AS_1_-transfected group was significantly higher than that in the tumor tissues of nude mice in the control group (Fig. 4e). Similarly, the expression of RhoC in the intraperitoneally injected nude mice in the lncRNA ABHD11-AS1-transfected group was significantly higher than that in the control group (Fig. 4g). Further, silencing of the RhoC gene in lncRNA ABHD11-AS_1_-transfected ovarian cancer cells inhibited the tumor-promoting effect of ABHD11-AS_1_ (Fig. 5a, *p* < 0.05), promoted apoptosis of the cancer cells (Fig. 5b, *p* < 0.05), and inhibited tumor metastasis (Fig. 5c, *p* < 0.05) and invasion (Fig. 5d *p* < 0.05). The protein expression of RhoC and its downstream targets P70s6k, MMP2, and BCL-xl also decreased as a result of RhoC gene silencing (Fig. 6). These results indicate that RhoC is a direct target of the lncRNA ABHD11-AS_1_.

## Discussion

Protein-coding genes have long been the focus of tumor studies. However, the present study shows that non-coding RNA, the lncRNA ABHD11-AS_1_ in particular, may also play an extensive and key role in regulating the molecular mechanism of tumors.

In the present study, we used qRT-PCR to show that the ABHD11-AS_1_ expression level was higher in ovarian cancer tissues than in normal ovarian tissue. The results of the study were in agreement with those of Lin [8, 9], who reported similar findings in gastric cancer tissue. In our study, the high expression of ABHD11-AS_1_ in the ovarian cancer cell lines A2780 and OVCAR3 was found to promote cancer cell proliferation, metastasis, and invasion, and inhibit cancer cell apoptosis. Moreover, silencing of the lncRNA ABHD11-AS_1_ resulted in a decrease in cancer cell proliferation, metastasis and invasion, and an increase in cancer cell apoptosis. We also found that high expression of ABHD11-AS_1_ could promote the in vivo tumor growth rate as well as intraperitoneal metastasis ability in nude mice. The above experiments suggest that the lncRNA ABHD11-AS_1_ plays a role in promoting cancer development and progression in ovarian cancer cells; however, the underlying mechanism is unclear.

Studies have shown that lncRNAs can combine with specific proteins, modulate the activity of the corresponding protein, and even alter cytoplasmic localization of the protein. Moreover, lncRNAs can also function as a structural component of protein nucleic acid complexes and thus play a role in cell differentiation, proliferation, and apoptosis, and consequently, disease progression [18–20]. In the present study, we have tried to determine whether the lncRNA ABHD11-AS_1_ affects tumor growth and progression via regulation of RhoC expression. RhoC was selected because it has been found to be expressed in a variety of tumors, such as liver, gastric, ovarian, and breast cancer; in addition, it is associated with the invasion and metastasis of tumors [21–31]. Rho signaling regulates tumor infiltration by adjusting the level of mucin E-calcium [32]; promotes invasion by regulation of MMP expression [33–35]; and promotes tumor angiogenesis, invasion and metastasis by promoting the expression of angiogenesis factors [36]. Our study found that high expression of the lncRNA ABHD11-AS_1_ in the ovarian cancer cell lines was associated with high expression of RhoC and its downstream molecules P70s6k, MMP2 and BCL-xL, while silencing of the lncRNA ABHD11-AS_1_ had the opposite effect. Thus, the findings indicate that the tumor-promoting effects of ABHD11-AS_1_ may be brought about via its effects on RhoC. Further, it seems that P70s6k, MMP2, and BCL-xL are downstream molecules of RhoC that are closely involved in the formation, metastasis and apoptosis of tumor cells.

We verified the involvement of RhoC in the mechanism of action of the lncRNA ABHD11-AS_1_ with the RNA pull-down experiment. Silencing of RhoC in stable lncRNA ABHD11-AS_1_-overexpressing ovarian cancer cells resulted in a decrease in cancer cell proliferation, invasion and metastasis and an increase in cancer cell apoptosis. Further, the protein expression of RhoC and its downstream proteins P70s6k, MMP2, BCL-xl also decreased. We also used immunohistochemical analysis to verify that high expression of the lncRNA ABHD11-AS_1_ in ovarian cancer cells in in vivo tumors in nude mice led to an increase in RhoC expression. All these findings together indicate that the lncRNA ABHD11-AS_1_ promotes the growth and metastasis of ovarian cancer cells by targeting RhoC.

LncRNAs can influence a variety of biological behaviors of tumor cells and may play an important role in the tumorigenesis and progression of tumors. The present study lays the foundation for future studies on the role of lncRNAs in tumor occurrence and progression. Investigating the molecular targets of lncRNAs will help identify new markers and targets for the diagnosis and treatment of cancers.

## Conclusion

This study is the first to prove that the lncRNA ABHD11-AS_1_ promotes ovarian cancer cell proliferation, invasion and metastasis, and inhibits ovarian cancer cell apoptosis by targeting RhoC and its downstream molecules. This finding has shed new light on the molecular mechanism underlying the occurrence and development of malignant ovarian tumors. Moreover, ABHD11-AS_1_ has now emerged as a potential new target for ovarian cancer treatment.

## Additional file

Additional file 1: Table 1. ABHD11-AS_1_ expression in normal ovary and ovarian carcinoma tissues. **Table 2.** Correlation of ABHD11-AS_1_ expression with different clinicopathological features of ovarian carcinoma. **Table 3.** The ABHD11-AS_1_ sequence was CTCGAGTGAAGACGGAAATGGGCGGGGCTGCGAGCTAGGGCGGGAGAAGGAGCGCGGGGAGGACGTACCTTGTGAGATGCGAGCCGGCCAACAGCTTGCAAGCATGCTCCGCTGGACCCGAGCCTGGAGGCTCCCGCGTGAGGGACTCGGCCCCCACGGCCCTAGCTTCGCGAGGGTGCCTGTCGCACCCAGCAGCAGCAGCGGCGGCCGAGGGGGCGCCGAGCCGAGGCCGCTTCCGCTTTCCTACAGGCTTCTGGACGGGGAGGCAGCCCTCCCGGCCGTCGTCTTTTTGCACGGGCTCTTCGGCAGCAAAACTAACTTCAACTCCATCGCCAAGATCTTGGCCCAGCAGACAGGCCGTGCTGACGGTGGATGCTCGTAACCACGGTGACAGCCCCCACAGCCCAGACATGAGCTACGAGATCATGAGCCAGGACCTGCAGGACCTTCTGCCCCAGCTGGGCCTGGTGCCCTGCGTCGTCGTTGGCCACAGCATGGGAGGAAAGACAGCCATGCTGCTGGCACTACAGAGGGTGAGCCGCCCATGTCTGGGGCCTCCTCCCATTCAGTATATACCCTGAGGGCCCTGCAGGCAACCTGGGACTCACATGATCGTTGGATGACCAAGTTCAGGCTCCAGGAGCCATGCCTGAGACTCCCTATGTCTGCCTAAGACTGGTCCCAGTTCGGTTCTCTCCCACAGCCAGAGCTGGTGGAACGTCTCATTGCTGTAGATATCAGCCCAGTGGAAAGCACAGGTGTCTCCCACTTTGCAACCTATGTGGCAGCCATGAGGGCCATCAACATCGCAGATGAGCTGCCCCGCTCCCGTGCCCGAAAACTGGCGGATGAACAGCTCAGTTCTGTCATCCAGGACATGGCCGTGCGGCAGCACCTGCTCACTAACCTGGTAGAGGTAGACGGGCGCTTCGTGTGGAGGGTGAACTTGGATGCCCTGACCCAGCACCTAGACAAGATCTTGGCTTTCCCACAGAGGCAGGAGTCCTACCTCGGGCCAACACTCTTTCTCCTTGGTGGAAACTCCCAGTTCGTGCATCCCAGCCACCACCCTGAGATTATGCGGCTCTTCCCTCGGGCCCAGATGCAGACGGTGCCGAACGCTGGCCACTGGATCCACGCTGACCGCCCACAGGACTTCATAGCTGCCATCCGAGGCTTCCTGGTCTAAGAGTTGCTGGCAAGAAGATGGCCGGGCGTGGTGGCTCATGCCTGTAATTCCAGCACTTTGGGAGGCTAAGGCGGGAGGATGACTTGAGGCCAGGAGTTGGAGACCAGCCTGGCCAACATGGTGAAACCCTGTCTCTACTAAAAATACAAAAATTAGCCTGGCGTGGTGGTGCACACCTGTAATCCCAGCTACTCTGGAGGCTGAGGCAGGAGAATCACTTGAACCCTGGAGGCAGAGGTTGCAATGAGCCGAGATCACACCACTACACTCCAGCCTAGGCAACAGAGCAAGACTCTGTCTCAAAAAAAACAAAACAAAAAGGAGGCACAAAACCCCAGGCTTCAAGTCTCTGCAGCCTGCTCCACATTTGGGCACAGAAGGACTCAGACAGGCACTGTGTGGGCACGAGGTTTTACAGGGGTGGTCAGACCTCAGGCTTTAATGAATAAAGACACTACTCCCAAAGGTACC. (DOCX 21 kb)

## Abbreviations

EOC: Epithelial ovarian carcinoma
MMP: Matrix metalloproteinase
P70S6K: P70 Ribosomal S6 kinase
qRT–PCR: Real-time quantitative PCR
RhoC: Ras homolog gene family member C
si-RNA: Small interfering RNA

## References

[1] Jayson GC, Kohn EC, Kitchener HC, Ledermann JA (2014). Ovarian cancer. Lancet.

[2] Yuan SX, Wang J, Yang F, Tao QF, Zhang J, Wang LL (2016). Long noncoding RNA DANCR increases stemness features of hepatocellular carcinoma by derepression of CTNNB1. Hepatology.

[3] Ma Y, Yang Y, Wang F, Moyer MP, Wei Q, Zhang P (2016). Long non-coding RNA CCAL regulates colorectal cancer progression by activating Wnt/β-catenin signalling pathway via suppression of activator protein 2α. Gut.

[4] Martens-Uzunova ES, Böttcher R, Croce CM, Jenster G, Visakorpi T, Calin GA (2014). Long noncoding RNA in prostate, bladder, and kidney cancer. Eur Urol.

[5] Fan J, Xing Y, Wen X, Jia R, Ni H, He J (2015). Long non-coding RNA ROR decoys gene-specific histone methylation to promote tumorigenesis. Genome Biol.

[6] Sun M, Kraus WL (2015). From discovery to function: the expanding roles of long non-coding RNAs in physiology and disease. Endocr Rev.

[7] Schaukowitch K, Kim TK (2014). Emerging epigenetic mechanisms of long non-coding RNAs. Neuroscience.

[8] Lin X, Yang M, Xia T, Guo J (2014). Increased expression of long noncoding RNA ABHD11-AS1in gastric cancer and its clinical significance. Med Oncol.

[9] Yang Y, Shao Y, Zhu M, Li Q, Yang F, Lu X (2016). Using gastric juice lncRNA-ABHD11-AS1 AS a novel type of biomarker in the screening of gastric cancer. Tumour Biol.

[10] Jaffe AB, Hall A (2005). Rho GTPases: biochemistry and biology. Annu Rev Cell Dev Biol.

[11] Zhao Y, Zheng HC, Chen S, Gou WF, Xiao LJ, Niu ZF (2013). The role of RhoC in ovarian epithelial carcinoma: a marker for carcinogenesis, progression, prognosis, and target therapy. Gynecol Oncol.

[12] Chen X, Chen S, Xiu YL, Sun KX, Zong ZH, Zhao Y (2015). RhoC is a major target of microRNA-93-5P in epithelial ovarian carcinoma tumorigenesis and progression. Mol Cancer.

[13] Lozano E, Betson M, Braga VM (2003). Tumor progression: small GTPases and loss of cell-cell adhesion. BioEssays.

[14] Czabotar PE, Lessene G, Strasser A, Adams JM (2014). Control of apoptosis by the BCL-2 protein family: implications for physiology and therapy. Nat Rev Mol Cell Biol.

[15] Rieger C, Huebner D, Temme A, Wirth MP, Fuessel S (2015). Antisense- and siRNA-mediated inhibition of the anti-apoptotic gene Bcl-xL for chemosensitization of bladder cancer cells. Int J Oncol.

[16] Bahrami-B F, Ataie-Kachoie P, Pourgholami MH, Morris DL (2014). p70 Ribosomal proteinS6 kinase (Rps6kb1): an update. J Clin Pathol.

[17] Ip CK, Yung S, Chan TM, Tsao SW, Wong AS (2014). p70 S6 kinase drives ovarian cancer metastasis through multicellular spheroid-peritoneum interaction and P-cadherin/b1 integrin signaling activation. Oncotarget.

[18] Wilusz JE, Sunwoo H, Spector DL (2009). Long noncoding RNAs: functional surprises from the RNA world. Genes Dev.

[19] Wang KC, Chang HY (2011). Molecular mechanisms of long noncoding RNAs. Mol Cell.

[20] Salmena L, Poliseno L, Tay Y, Kats L, Pandolfi PP (2011). A ceRNA hypothesis: the rosetta stone of a hidden RNA language. Cell.

[21] Liu N, Zhang G, Bi F, Pan Y, Xue Y, Shi Y (2007). RhoC is essential for the metastasis of gastric cancer. J Mol Med (Berl).

[22] Hakem A, Sanchez-Sweatman O, You-Ten A, Duncan G, Wakeham A, Khokha R, et al. RhoC is dispensable for embryogenesis and tumor initiation but essential for metastasis. Genes Dev. 2005;19:1974–79. 10.1101/gad.1310805 PMC1199568 16107613

[23] Kleer CG, van Golen KL, Zhang Y, Wu ZF, Rubin MA, Merajver SD (2002). Characterization of RhoC expression in benign and malignant breast disease: a potential new marker for small breast carcinomas with metastatic ability. Am J Pathol.

[24] van Golen KL, Wu ZF, Qiao XT, Bao LW, Merajver SD (2000). RhoC GTPase, a novel transforming oncogene for human mammary epithelial cells that partially recapitulates the inflammatory breast cancer phenotype. Cancer Res.

[25] Islam M, Lin G, Brenner JC, Pan Q, Merajver SD, Hou Y (2009). RhoC expression and head and neck cancer metastasis. Mol Cancer Res.

[26] Kleer CG, Teknos TN, Islam M, Marcus B, Lee JS, Pan Q (2006). RhoC GTPase expression as a potential marker of lymph node metastasis in squamous cell carcinomas of the head and neck. Clin Cancer Res.

[27] Kondo T, Sentani K, Oue N, Yoshida K, Nakayama H, Yasui W (2004). Expression of RhoC is associated with metastasis of gastric carcinomas. Pathobiology.

[28] Wang W, Wu F, Fang F, Tao Y, Yang L (2008). Inhibition of invasion and metastasis of hepatocellular carcinoma cells via targeting RhoC in vitroand in vivo. Clin Cancer Res.

[29] Yao H, Dashner EJ, van Golen CM, van Golen KL (2006). RhoC GTPase is required for PC-3 prostate cancer cell invasion but not motility. Oncogene.

[30] Sequeira L, Dubyk CW, Riesenberger TA, Cooper CR, van Golen KL (2008). Rho GTPases in PC-3 prostate cancer cell morphology, invasion and tumor cell diapedesis. Clin Exp Metastasis.

[31] Faried A, Faried LS, Kimura H, Nakajima M, Sohda M, Miyazaki T (2006). RhoA and RhoC proteins promote both cell proliferation and cell invasion of human oesophageal squamous cell carcinoma cell lines in vitroandin vivo. Eur J Cancer.

[32] Xia L, Huang W, Tian D, Zhu H, Zhang Y, Hu H (2012). Upregulated FoxM1 expression induced by hepatitis B virus X protein promotes tumor metastasis and indicates poor prognosis in hepatitis B virus-related hepatocellular carcinoma. J Hepatol.

[33] Liu X, Wang C, Chen Z (2011). MicroRNA-138 suppresses epithelial-mesenchymal transition in squamous cell carcinoma cell lines. Biochem J.

[34] Xue G, Zou X, Zhou JY, Sun W, Wu J, Xu JL (2013). Raddeanin a induces human gastric cancer cells apoptosis and inhibits their invasion in vitro. Biochem Biophys Res Commun.

[35] Bradford CR, Kumar B, Bellile E, Lee J, Taylor J, D'Silva N (2014). Biomarkers in advanced larynx cancer. Laryngoscope.

[36] Nakabayashi H, Shimizu K (2011). HA1077, a rho kinase inhibitor,suppresses glioma-induced angiogenesis by targeting the rho-ROCK and the mitogen-activated protein kinase kinase/extracellular signal-regulatedkinase (MEK/ERK) signal pathways. Cancer Sci.

