# Supplementary material for: Role of the lncRNA ABHD11-AS1 in the tumorigenesis and progression of epithelial ovarian cancer through targeted regulation of RhoC
Source: Mol Cancer. 2017 Aug 17;16:138. doi: 10.1186/s12943-017-0709-5 (PMC5561620; doi:10.1186/s12943-017-0709-5)
Supplement: Additional file 1: Table 1. — ABHD11-AS1 expression in normal ovary and ovarian carcinoma tissues. Table 2. Correlation of ABHD11-AS1 expression with different clinicopathological features of ovarian carcinoma. Table 3. The ABHD11-AS1 sequence was CTCGAGTGAAGACGGAAATGGGCGGGGCTGCGAGCTAGGGCGGGAGAAGGAGCGCGGGGAGGACGTACCTTGTGAGATGCGAGCCGGCCAACAGCTTGCAAGCATGCTCCGCTGGACCCGAGCCTGGAGGCTCCCGCGTGAGGGACTCGGCCCCCACGGCCCTAGCTTCGCGAGGGTGCCTGTCGCACCCAGCAGCAGCAGCGGCGGCCGAGGGGGCGCCGAGCCGAGGCCGCTTCCGCTTTCCTACAGGCTTCTGGACGGGGAGGCAGCCCTCCCGGCCGTCGTCTTTTTGCACGGGCTCTTCGGCAGCAAAACTAACTTCAACTCCATCGCCAAGATCTTGGCCCAGCAGACAGGCCGTGCTGACGGTGGATGCTCGTAACCACGGTGACAGCCCCCACAGCCCAGACATGAGCTACGAGATCATGAGCCAGGACCTGCAGGACCTTCTGCCCCAGCTGGGCCTGGTGCCCTGCGTCGTCGTTGGCCACAGCATGGGAGGAAAGACAGCCATGCTGCTGGCACTACAGAGGGTGAGCCGCCCATGTCTGGGGCCTCCTCCCATTCAGTATATACCCTGAGGGCCCTGCAGGCAACCTGGGACTCACATGATCGTTGGATGACCAAGTTCAGGCTCCAGGAGCCATGCCTGAGACTCCCTATGTCTGCCTAAGACTGGTCCCAGTTCGGTTCTCTCCCACAGCCAGAGCTGGTGGAACGTCTCATTGCTGTAGATATCAGCCCAGTGGAAAGCACAGGTGTCTCCCACTTTGCAACCTATGTGGCAGCCATGAGGGCCATCAACATCGCAGATGAGCTGCCCCGCTCCCGTGCCCGAAAACTGGCGGATGAACAGCTCAGTTCTGTCATCCAGGACATGGCCGTGCGGCAGCACCTGCTCACTAACCTGGTAGAGGTAGACGGGCGCTTCGTGTGGAGGGTGAACTTGGATGCCCTGACCCAGCACCTAGACAAGATCTTGGCTTTCCCACAGAGGCAGGAGTCCTACCTCGGGCCAACACTCTTTCTCCTTGGTGGAAACTCCCAGTTCGTGCATCCCAGCCACCACCCTGAGATTATGCGGCTCTTCCCTCGGGCCCAGATGCAGACGGTGCCGAACGCTGGCCACTGGATCCACGCTGACCGCCCACAGGACTTCATAGCTGCCATCCGAGGCTTCCTGGTCTAAGAGTTGCTGGCAAGAAGATGGCCGGGCGTGGTGGCTCATGCCTGTAATTCCAGCACTTTGGGAGGCTAAGGCGGGAGGATGACTTGAGGCCAGGAGTTGGAGACCAGCCTGGCCAACATGGTGAAACCCTGTCTCTACTAAAAATACAAAAATTAGCCTGGCGTGGTGGTGCACACCTGTAATCCCAGCTACTCTGGAGGCTGAGGCAGGAGAATCACTTGAACCCTGGAGGCAGAGGTTGCAATGAGCCGAGATCACACCACTACACTCCAGCCTAGGCAACAGAGCAAGACTCTGTCTCAAAAAAAACAAAACAAAAAGGAGGCACAAAACCCCAGGCTTCAAGTCTCTGCAGCCTGCTCCACATTTGGGCACAGAAGGACTCAGACAGGCACTGTGTGGGCACGAGGTTTTACAGGGGTGGTCAGACCTCAGGCTTTAATGAATAAAGACACTACTCCCAAAGGTACC. (DOCX 21 kb) [file 12943_2017_709_MOESM1_ESM.docx]

**Supplementary Table 1:** ABHD11-AS_1_ expression in normal ovary and ovarian carcinoma tissues

| **Groups** | **N** | **ABHD11-AS_1_ expression / 18s** | ***P* value** |
| --- | --- | --- | --- |
|  |  |  |  |
| Normal ovary | 13 | 1.25E-06 ± 1.60E-06 | ***0.0271*** |
| Ovarian carcinoma | 51 | 1.32E-05 ± 4.33E-05 |  |

Bold and Italics means P < 0.05.

**Supplementary Table 2:** Correlation of ABHD11-AS_1_ expression with different clinicopathological features of ovarian carcinoma

| **Clinicopathological features** | **N** | **ABHD11-AS_1_ expression / 18s** | ***P* value** |
| --- | --- | --- | --- |
|  |  |  |  |
| **The pathology types** |  |  | 0.090 |
| Serous carcinoma | 46 | 1.42E-05 ±4.55E-05 |  |
| The other pathology types | 5 | 4.13E-06 ±7.04E-06 |  |
| **Age** |  |  | 0.271 |
| ≤ 52 | 27 | 9.70E-06 ± 3.49E-05 |  |
| > 52 | 24 | 1.72E-05 ± 5.16E-05 |  |
| **FIGO stages** |  |  | ***0.048*** |
| I-II | 15 | 2.92E-06 ± 4.23E-06 |  |
| III-IV | 36 | 1.75E-05 ± 5.11E-05 |  |
| **Pathology classification** |  |  |  |
| Well | 10 | 1.96E-06 ± 2.03E-06 |  |
| Mod + Poor | 41 | 1.60E-05± 4.80E-05 | ***0.034*** |
| Bold and Italics means P < 0.05. | | | |

**Supplementary Table 3:** The ABHD11-AS_1_ sequence was CTCGAGTGAAGACGGAAATGGGCGGGGCTGCGAGCTAGGGCGGGAGAAGGAGCGCGGGGAGGACGTACCTTGTGAGATGCGAGCCGGCCAACAGCTTGCAAGCATGCTCCGCTGGACCCGAGCCTGGAGGCTCCCGCGTGAGGGACTCGGCCCCCACGGCCCTAGCTTCGCGAGGGTGCCTGTCGCACCCAGCAGCAGCAGCGGCGGCCGAGGGGGCGCCGAGCCGAGGCCGCTTCCGCTTTCCTACAGGCTTCTGGACGGGGAGGCAGCCCTCCCGGCCGTCGTCTTTTTGCACGGGCTCTTCGGCAGCAAAACTAACTTCAACTCCATCGCCAAGATCTTGGCCCAGCAGACAGGCCGTGCTGACGGTGGATGCTCGTAACCACGGTGACAGCCCCCACAGCCCAGACATGAGCTACGAGATCATGAGCCAGGACCTGCAGGACCTTCTGCCCCAGCTGGGCCTGGTGCCCTGCGTCGTCGTTGGCCACAGCATGGGAGGAAAGACAGCCATGCTGCTGGCACTACAGAGGGTGAGCCGCCCATGTCTGGGGCCTCCTCCCATTCAGTATATACCCTGAGGGCCCTGCAGGCAACCTGGGACTCACATGATCGTTGGATGACCAAGTTCAGGCTCCAGGAGCCATGCCTGAGACTCCCTATGTCTGCCTAAGACTGGTCCCAGTTCGGTTCTCTCCCACAGCCAGAGCTGGTGGAACGTCTCATTGCTGTAGATATCAGCCCAGTGGAAAGCACAGGTGTCTCCCACTTTGCAACCTATGTGGCAGCCATGAGGGCCATCAACATCGCAGATGAGCTGCCCCGCTCCCGTGCCCGAAAACTGGCGGATGAACAGCTCAGTTCTGTCATCCAGGACATGGCCGTGCGGCAGCACCTGCTCACTAACCTGGTAGAGGTAGACGGGCGCTTCGTGTGGAGGGTGAACTTGGATGCCCTGACCCAGCACCTAGACAAGATCTTGGCTTTCCCACAGAGGCAGGAGTCCTACCTCGGGCCAACACTCTTTCTCCTTGGTGGAAACTCCCAGTTCGTGCATCCCAGCCACCACCCTGAGATTATGCGGCTCTTCCCTCGGGCCCAGATGCAGACGGTGCCGAACGCTGGCCACTGGATCCACGCTGACCGCCCACAGGACTTCATAGCTGCCATCCGAGGCTTCCTGGTCTAAGAGTTGCTGGCAAGAAGATGGCCGGGCGTGGTGGCTCATGCCTGTAATTCCAGCACTTTGGGAGGCTAAGGCGGGAGGATGACTTGAGGCCAGGAGTTGGAGACCAGCCTGGCCAACATGGTGAAACCCTGTCTCTACTAAAAATACAAAAATTAGCCTGGCGTGGTGGTGCACACCTGTAATCCCAGCTACTCTGGAGGCTGAGGCAGGAGAATCACTTGAACCCTGGAGGCAGAGGTTGCAATGAGCCGAGATCACACCACTACACTCCAGCCTAGGCAACAGAGCAAGACTCTGTCTCAAAAAAAACAAAACAAAAAGGAGGCACAAAACCCCAGGCTTCAAGTCTCTGCAGCCTGCTCCACATTTGGGCACAGAAGGACTCAGACAGGCACTGTGTGGGCACGAGGTTTTACAGGGGTGGTCAGACCTCAGGCTTTAATGAATAAAGACACTACTCCCAAAGGTACC.
